# Worldwide Occurrence and Investigations of Contamination of Herbal Medicines by Tropane Alkaloids

**DOI:** 10.3390/toxins9090284

**Published:** 2017-09-15

**Authors:** Thomas Y. K. Chan

**Affiliations:** 1 Division of Clinical Pharmacology and Drug and Poisons Information Bureau, Department of Medicine and Therapeutics, Faculty of Medicine, The Chinese University of Hong Kong, Prince of Wales Hospital, Shatin, New Territories, Hong Kong, China; tykchan@cuhk.edu.hk; Tel.: +852-3505-3907; 2 Prince of Wales Hospital Poison Treatment Centre, Hong Kong, China

**Keywords:** tropane alkaloids, anticholinergic poisoning, herbal medicines, medicinal plants

## Abstract

Tropane alkaloids occur mainly in *Solanaceae* plants. In the present review, the main objective is to describe the worldwide occurrence and investigations of anticholinergic poisoning due to the contamination of herbal teas and herbs by tropane alkaloids. Tropane alkaloid poisoning can occur after consumption of any medicinal plant if *Solanaceae* plants or plant parts are present as contaminants. Globally, almost all reports in 1978–2014 involve herbal teas and one of the prescribed herbs in composite formulae. Contamination most likely occurs during harvest or processing. As for prescribed herbs, on-site inspection is necessary to exclude cross-contamination and accidental mix-up at the retail level. The diagnosis is confirmed by screening for the presence of *Solanaceae* species and tropane alkaloids. Herbal teas and herbs contaminated by tropane alkaloids can pose a serious health hazard because these relatively heat-stable alkaloids may exist in large quantities. The WHO repeatedly emphasises the importance of good agricultural and collection practices for medicinal plants. DNA barcoding is increasingly used to exclude the presence of contaminants (particularly toxic species) and product substitution. All suspected cases should be reported to health authorities so that investigations along the supply chain and early intervention measures to protect the public can be initiated.

## 1. Introduction

**Figure 1:**
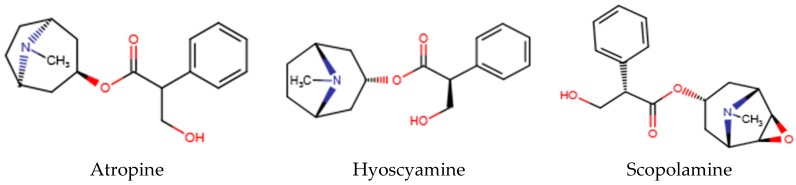
Atropine (racemic mixture of (-)-hyoscyamine and (+)-hyoscyamine), hyoscyamine and scopolamine (also known as hyoscine) are the most important tropane alkaloids.

Tropane alkaloids (e.g., atropine, hyoscyamine, and scopolamine, Figure 1) occur mainly in the plants of the *Solanaceae* genus, particularly in species that have long been known for their medicinal, toxic, and hallucinogenic properties [1,2,3]. These plants include: *Datura metel, D. innoxia, D. ferox*, *D. stramonium* (Jimson weed or thorn apple)*, Hyoscyamus niger* (henbane)*, Atropa mandragora* (mandrake), and *A. belladonna* (deadly nightshade). Tropane alkaloids are also found in other *Angiosperm* families, namely *Brassicaceae*, *Convolvulaceae* (e.g., *Erycibe henryi*), *Erythroxylaceae*, *Euphorbiaceae*, *Proteaceae*, and *Rhizophoraceae* [3].

The toxicity (anticholinergic effects) of *Solanaceae* plants can be attributed to their tropane alkaloids content and profile (i.e., the relative amount of atropine, hyoscyamine, and scopolamine), which can differ greatly between species, geographical regions, and harvesting stages [3]. All parts of the plants are toxic, including the flowers, seeds (fruits), leaves, and stems.

Exposures to tropane alkaloids, as reported to American Poison Control Centres [4], are mostly accidental, mainly involving children. Increasingly common is the deliberate ingestion of the *Datura* species for their psychoactive properties [2,3]. Intentional ingestion for self-harm and improper use of *Solanaceae* plants for medicinal purposes also increase the risk of poisoning [4,5].

Unintended ingestion of tropane alkaloids may also occur because of the contamination of food or a mix-up of edible (parts of) plants with *Solanaceae* plants [3,6]. The problem with food contamination can be widespread, as indicated by a pan-Europe survey. When food samples collected in 2015 were tested for chemical contaminants, 2343 (11.2%) of 20,885 analytical results were positive for tropane alkaloids [7]. Fortunately, there were comparatively few poisoning incidents due to contamination of food by tropane alkaloids [3,6].

Similarly, contamination, erroneous substitution, and a mix-up of herbal teas and herbs with *Solanaceae* plants result in exposures to tropane alkaloids [1,5,6]. For example, in traditional Chinese medicine, the dried flowers of *D. metel* are used to treat asthma, chronic bronchitis, pains, and other conditions [1]. As the recommended dose (0.3–0.6 g) per decoction is generally followed, poisoning rarely happens. Due to their very similar appearances, *Flos Campsis* can be erroneously substituted by *D. metel* [5]. Overdose then occurs, because *D. metel* will be dispensed according to the prescribed dose of the intended *Flos Campsis* (5–9 g). In the case of contamination of herbal teas and herbs, the quantities of tropane alkaloids present can be dangerously high [5,6].

In this review, the main objective is to describe the worldwide occurrence and investigations of anticholinergic poisoning due to the contamination of herbal medicines by tropane alkaloids. Reports of contamination of plants used for medicinal purposes (i.e., herbal teas and herbs) are included. Herbal teas are freely available. On the other hand, herbs in composite formulae are typically used with supervision from herbalists, and the use of all potent herbs [8] should be regulated.

## 2. Methodology

To identify journal articles on tropane alkaloid poisoning related to the use of herbal medicines (herbs and herbal teas), a search of Medline (1975–July 2017) and Wanfang Data (1998–July 2017) was performed, using anticholinergic and tropane alkaloids as the keywords. Additional journal articles and other publications were identified by searching Google, Google Scholar, and the herbal medicine database of our Drug and Poisons Information Bureau [9,10]. This database is continuously updated by a multidisciplinary team to include the latest information on important medicinal plants.

This review includes all published reports of tropane alkaloid poisoning caused by the contamination of non-toxic medicinal plants, used alone (in herbal teas), in proprietary preparations (as herbal slimming aids), or together with other herbs (in composite formulae as herbal decoctions). In all situations, the investigation results were reviewed to determine the reasons for the contamination of non-toxic medicinal plants by tropane alkaloids and possible preventive measures. Redundant publications and poisoning cases due to plant misidentification or erroneous substitution [5] were excluded.

Since only published data and information freely available in the public domain were reviewed, research ethics committee approval was not required.

## 3. Results

Reports on the contamination of herbal teas (Table 1) and herbs in proprietary preparations (Table 2) or composite formulae (Table 3) resulting in anticholinergic poisoning were presented separately because the medicinal plants involved, the situations of their use, and the case complexity were quite different. Since the medicinal plants themselves are non-toxic, there must have been contamination by foreign matters (plants or plant parts) containing tropane alkaloids.

From 1978 to 2013, there were 10 reports of tropane alkaloid poisoning due to the contamination of herbal teas [11,12,13,14,15,16,17,18,19,20]. These reports originated from the USA (*n* = 3), UK (*n* = 2), Austria (*n* = 1), Canada (*n* = 1), France (*n* = 1), Netherlands (*n* = 1), and Spain (*n* = 1) (Table 1).

The contamination of seven types of herbal teas had been reported: burdock root (*Arctium lappa*) (*n* = 2), comfrey (*Symphytum officinale*) (*n* = 2), common mallow *(Malva sylvestris)* (*n* = 1), lungwort *(Pulmonaria officinalis)* (*n* = 1) marshmallow root *(Althaea officinalis)* (*n* = 1), stinging nettle (*Urtica dioica*) (*n* = 1), and paraguay tea (*Ilex paraguariensis*) (*n* = 1).

Herbal teas obtained from the patients were found to contain impurities (*A. belladonna* berries or leaves) [15,20] or tropane alkaloids [11,12,14,16,17,19]. It was noted that the foreign matters in herbal teas could contain atropine as well as hyoscyamine and scopolamine [19]. In four reports, the atropine contents ranged from 0.76 to >30 mg per g herbal tea [11,12,16,17], but the quantities of hyoscyamine and scopolamine present, if any, were not measured. In the early studies, this was in part because of the limitations in the analytical techniques [15]. In one report, the herbal tea was estimated by in vitro study to contain atropine ≥0.14 mg per g [14]. In two reports, a batch strongly suspected or known to be contaminated with tropane alkaloids was consumed [13,18]. In two reports, the investigations along the production chain were described [16,17], with contamination by *A. belladonna* probably occurring during harvest.

Each poisoning incident involved one to seven subjects, and altogether 25 subjects presented with mild to severe anticholinergic toxicity. At least 10 subjects needed hospitalisation, and 10 other subjects needed emergency department attendance.

In 2005, three cases of serious anticholinergic poisoning were reported from Italy, following the use of proprietary slimming products containing *Coleus forskohlii* [21] (Table 2). The clustering of cases after the use of herbal products containing the same ingredient (*C. forskohlii*), and the unexplained occurrence of anticholinergic toxicity necessitated urgent investigations into their quality [22]. All the products involved were from the same batch of *C. forskohlii*, which was contaminated by tropane alkaloids. This outbreak highlighted the importance of quality control of medicinal plants, especially their proprietary preparations. At least two of the three affected subjects needed hospitalisation.

From 1985 to 2014, there were 11 reports of tropane alkaloid poisoning caused by the contamination of one of the dozen herbs prescribed by a herbalist [23,24,25,26,27,28,29,30,31,32]. The reports came from Hong Kong (*n* = 4) or China (*n* = 7), where traditional Chinese medicine is an integral part of the healthcare systems and there are well-established, territory-wide systems to monitor the safety and quality of herbs [9,33]. In Table 3, the case series and herbs most frequently involved are listed first.

In two reports, the non-toxic herbs with contaminants could not be identified, although the herbal samples obtained from one [23] or all [24] of the patients were tested positive for atropine [23] or tropane alkaloids (unspecified) [24].

In nine reports [24,25,26,27,28,29,30,31,32], the contaminated herbs were successfully identified—*Rhizoma Atractylodis* (*n* = 6), *Radix Aucklandiae* (*n* = 1), *Radix Strobilanthis Forrestii,* (*n* = 1), and *Rhizoma et Radix Notopterygii* (*n* = 1). Firstly, morphological examination revealed the presence of rhizomes [27,29,30], seeds [25], or impurities [25] originating from *Solanaceae* plants. Secondly, tropane alkaloids were present in the offending herb [24,27,30,31,32], and there was a marked decrease in the incidence of anticholinergic poisoning immediately after its withdrawal [28]. However, even if the tropane alkaloids present were specified [25,27,31], their relative abundance was not mentioned.

By far the most extensive experience was shared by the Department of Health, Hong Kong [26], based on their investigations of 11 poisoning incidents involving contaminated *Rhizoma Atractylodis* during 2002–2011. It was likely that contamination by tropane alkaloids occurred upstream of the supply chain, since the batches implicated were pre-packed and supplied intact from the exporters.

Each poisoning incident involved one to >10 subjects. Available information indicated that most of these affected subjects needed hospitalisation [24,25,26,27,30,31,32].

All the cases with tropane alkaloid poisoning as summarised in Table 1, Table 2 and Table 3 made a full recovery. Most of the subjects required hospital treatment.

## 4. Discussion

Anticholinergic poisoning caused by the contamination of medicinal plants should be a high priority for early detection and containment, since potent alkaloids capable of causing severe toxicity and even death are involved [2,5]. Tropane alkaloids are fairly heat-stable [3]. Hence, the brewing of herbal teas and boiling of herbs cannot provide protection. Tropane alkaloids compete with acetylcholine for binding sites on the muscarinic receptors, producing both peripheral (e.g., dilated pupils, blurred vision, hyperthermia, dry mouth, dry flushed skin, tachycardia, reduced bowel motility, and urinary retention) and central (e.g., hallucinations, delirium, drowsiness, amnesia, ataxia, seizures, and coma) antimuscarinic effects [2,5]. Their toxicity is dose-dependent, with more profound effects as the dose increases. For adults, an oral dose of 0.5 mg atropine will produce acute side effects (slight cardiac slowing, some dryness of mouth, and inhibition of sweating); an oral dose of 1 mg atropine causes definite dryness of mouth, thirst, acceleration of heart rate, and mild dilation of pupils [3]. Hyoscyamine has about twice the antimuscarinic activity of atropine [3]. Scopolamine is a more powerful suppressant of salivation, mydriatic, and hallucinogen than atropine [2,3]. Infants and young children are especially sensitive to the toxic effects of any anticholinergics; they will also be exposed to a greater amount per kg body weight [2,34].

Primary prevention is the best approach to all toxic exposures. The extent of the problem and possible reasons for contamination should be defined first after studying the worldwide reports in a systematic manner. Such information will facilitate the planning of preventive measures. Previous studies and surveys on food contamination by tropane alkaloids should also provide some insight into the problem, since good agricultural and collection practices are equally important and the harvest and processing of plants are also involved.

It has been known for decades that the contamination of food and herbal medicines with tropane alkaloid-containing plant species can occur [1,3]. Regarding anticholinergic toxicity associated with food contamination, a review by the European Food Safety Authority Panel on Contaminants in the Food Chain clearly identifies the presence of *D. stramonium* seeds as the most common problem [3]. This has been reported in the food poisoning incidents in Turkey (flour, 1949), Ethiopia (corn, 1992), Botswana (sorghum flour, 1998), Slovenia (buckwheat flour, 2003), and Austria (whole millet grain, 2006) [35,36,37,38,39]. Impurities that are less frequently found in food include seeds from other *Datura* species, *H. niger* seeds, and *A. belladonna* berries [3,6]. During 2015–2016, 1709 samples of plant-derived food products, mainly produced in Europe, were collected from retail stores in nine European countries and analysed for tropane alkaloids [40]. One or more tropane alkaloids were detected in 21.3% of single component flours, 20.0% of cereal-based food for young children (6–36 months), 6.8% of breakfast cereals, 14.6% of biscuits and pastry, 15.8% of bread, 26.2% of legumes and stir-fry mixes, 100% of potatoes, and 92.7% of aubergines. The highest mean tropane alkaloid concentration was detected in cereal-based meals for young children (130.7 μg/kg). Atropine and scopolamine were the most frequently detected. It is generally believed that food contamination occurs when toxic plants (parts) are accidentally mixed with edible plants during harvest or processing [6]. The emerging potential for food contamination from non-food weed plants that are encroaching on field crops in Europe is also highlighted [40].

In comparison, much less is known about the contamination of medicinal plants (especially herbs) by tropane alkaloids. Despite a well-structured and extensive literature search [5,9], very few reports could be identified (Table 1, Table 2 and Table 3). These herbal poisoning might be under-reported or under-recognised for several reasons. There was a lack of expertise in handling a complex case of herbal toxicity [10], such as reviews of written prescriptions, morphological examination of herbs, and toxicological analyses of urine and herbal residues. The non-toxic herb implicated and the impurities responsible could not be identified. Even if there was a nationwide channel for reporting [9], the findings were either unpublished or inaccessible. Isolated reports, especially without morphological or toxicological laboratory confirmation, were not publishable in medical journals.

The worldwide reports from 1978 to 2013 indicated that tropane alkaloid poisoning could occur after consumption of a contaminated herbal tea (Table 1). As the identity of the only medicinal plant in the commercially packaged herbal tea and its pharmacological effects were known, the diagnosis of anticholinergic poisoning caused by contaminants should be straightforward, with confirmation by screening for the presence of *Solanaceae* species and tropane alkaloids. Of particular concern was the presence of very high atropine content in one herbal tea sample (>30 mg per g) [11], in comparison with the doses that are associated with mild symptoms (0.5–1 mg) [3]. Contamination of herbal teas probably occurred during harvest [17]. Warning to the public and the immediate withdrawal of the contaminated batch from the market would help prevent further attacks. In The Netherlands, the poisoning incident involving contaminated marshmallow root [17] well illustrated the importance of such intervention measures and poison centre networking.

In recent years, surveys were conducted on the presence of tropane alkaloids in herbal teas. In Israel, 40 pre-packed tea bags of five different types of herbal teas produced by four international brands were collected from supermarkets in 2015 [41]. The atropine and scopolamine occurrence and levels were as follows: peppermint (80%, 20–208 and 20–208 µg/kg), fennel (20%, 83 and 11 µg/kg), rooibos (10%, 0 and 2 µg/kg), chamomile (10%, 0 and 2.1 µg/kg), and melissa (0%). The per tea bag tropane alkaloid levels were below the Acute Reference Dose (ARfD) (0.016 μg/kg body weight), expressed as the sum of hyoscyamine and scopolamine, assuming equivalent potency. The ARfD is defined by the European Food Safety Authority as a dose which, upon ingestion over a short period of time, does not pose appreciable health risks to humans [3]. In Europe, the 2015–2016 survey reported tropane alkaloid occurrence in 70.2% of 121 herbal teas products (75% of unknown country of origin, 65% classified as mixed herbal teas), with levels ranging from 71.4 to 4357.6 µg/kg [40]. Atropine (60%) and scopolamine (53%) were the contaminants most commonly found. Convolvine (found in the *Convolvulaceae* family) contributed most (72%) to the mean total concentration of tropane alkaloids, followed by atropine, scopolamine, and tropine (altogether 13.5%). Using boiling water, the efficiency of the transfer of tropane alkaloids (levels ≥10 μg/kg) from dry products to herbal tea infusions varied (atropine 54%, scopolamine 42%, and convolvine 23%). The sources and routes of contamination were still largely unknown [40].

Rarely, anticholinergic poisoning could occur after exposure to a contaminated medicinal plant in a proprietary preparation used for weight reduction (Table 2). In fact, herbal anti-obesity products can be very heterogeneous in nature [42]. Adverse effects of some products can be attributed to the intrinsic toxicity of the herbs and adulterants. The small cluster of cases in Italy [21] reminded us that a proprietary preparation with non-toxic herbs can turn toxic because of the unexpected presence of tropane alkaloids as contaminants. Quality control in the sourcing and processing of the herbs is of obvious importance.

The third situation where the public could be exposed to a contaminated medicinal plant, as reviewed by the worldwide reports from 1989 to 2013 (Table 3), was through the consumption of a non-toxic herb among the dozen items prescribed for an herbal decoction. It was a major challenge, even for a multidisciplinary team [10], to identify the offending herb. Although contamination of some commonly used herbs was first reported in the 1980s [23], >10 years had passed before the second report, to alert the healthcare professionals, appeared [24]. The reports mostly provided very brief details about the investigation process and main findings. The approach by the Department of Health, Hong Kong was described elsewhere [5]. Careful review of the herbal formulae for herbs over-represented or implicated in previous reports provided the first clue about the target for investigations. Morphological examination of the pack of unused herbs might identify impurities or plant parts from *Solanaceae* species. Such foreign matters and contaminated herbs would test positive for tropane alkaloids. On-site inspection and pre-packed stock supplied intact should exclude mix-up with toxic herbs and contamination at the retail level. Investigations along the supply chain should help establish the extent of the problem and reasons for the contamination by tropane alkaloids. If indicated, the health authority of the exporting country was notified to investigate the upstream of the supply chain. In China, similar investigations at the clinic, retail, or manufacturing levels were conducted. Based on the investigation findings, several conclusions could be made. The plant most commonly involved was *Rhizoma Atractylodis*. Morphological examination could reveal the presence of rhizomes, seeds, and impurities from *Solanaceae* plants. Investigations along the supply chain together with preventive measures to protect the public were necessary.

Available information, based on the investigations of poisoning incidents (Table 1, Table 2 and Table 3) and surveys of products from the market [40,41] indicates that the contamination of a medicinal plant most likely occurs during harvest or processing. However, for medicinal plants that are used in a composite formula (Table 3), the possibility of cross-contamination at the retailer shop should be excluded [5]. The WHO repeatedly emphasises the importance of good agricultural and collection practices for medicinal plants, with practical suggestions on how to avoid contamination along the production chain [43]. The considerable disparity between knowledge and implementation and the importance of training for farmers, producers, handlers, and processors to follow good practices are well recognised [43].

Depending on the expertise and resources available, taxonomy, herbarium, macroscopic and microscopic examination, and chemical analyses (bioactive compounds and chemical fingerprints) are used to identify medicinal plants [10]. However, dried, processed, or damaged plant parts will be difficult to identify. In the past decade, DNA barcoding is increasingly used in the quality control of herbal medicines to exclude the presence of contaminants (particularly toxic plant species) and product substitution [44,45]. This involves the use of a standardised region of DNA (DNA barcode) for taxonomic identification and the characterisation of plant species. If the results indicate that the species is different from the intended plant, the use of this sample, and even the entire batch, should be avoided. A DNA barcode database for medicinal plants has been established. This contains 78,847 barcodes from 23,262 medicinal species listed in the Chinese, European, Indian, Japanese, Korean, and American Herbal Pharmacopoeias [45].

Herbal teas and herbs contamination by these potent alkaloids can pose a serious health hazard because of their possible existence in large amounts and relative stability to heat. All suspected cases should be reported to the health authorities so that intervention measures to protect the public can be initiated immediately. Investigations along the supply chain to define the extent of the problem and the reasons of contamination are needed. Especially for poisoning of public health significance, national surveillance system for early detection and good collaboration among poison centres and health authorities are essential to facilitate risk management and swift action plans.

## 5. Conclusions

Tropane alkaloid poisoning can occur after consumption of any medicinal plant if *Solanaceae* plants or plant parts are present as contaminants. Globally, almost all reports involve herbal teas and one of the prescribed herbs in composite formulae. Contamination most likely occurs during harvest or processing. The WHO repeatedly emphasises the importance of good agricultural and collection practices for medicinal plants, with practical suggestions on how to avoid contamination along the production chain. As for prescribed herbs, on-site inspection should exclude accidental mix-up and cross-contamination at the retail level. DNA barcoding is increasingly used to exclude the presence of contaminants (particularly the toxic species) and product substitution in medicinal plants. When anticholinergic poisoning caused by contaminated medicinal plants is suspected, the presence of *Solanaceae* species and tropane alkaloids should be excluded. Herbal teas and herbs contaminated by tropane alkaloids can pose a serious health hazard because these relatively heat-stable alkaloids may exist in large quantities. Not surprisingly, affected subjects very often require hospitalisation. All suspected cases should be reported to the health authorities so that investigations along the supply chain and early intervention measures to protect the public can be initiated.

## Figures and Tables

**Table 1:** Tropane alkaloid poisoning caused by the contamination of herbal teas.

Species	Place, Year ^a^ [ref]	Details
Burdock root tea	USA, 1978 [11]	F/26 had mild symptoms after the first dose and needed ED attendance after the second (bigger) dose; her husband had mild symptoms after the first dose; commercial preparation obtained from the patient contained atropine >30 mg per g
Burdock root tea	USA, 1984 [12]	F/59 needed ED attendance; commercial preparation provided by the patient contained 0.76 mg atropine per g
Comfrey tea	UK, 1983 [13]	An elderly man needed hospital admission; his wife was cared for at home; their batch was known to be contaminated with *A. belladonna*
Comfrey tea	UK, 1989 [14]	M/30 needed hospital admission; tea leaves estimated by in vitro study were shown to contain ≥4 mg per 28 g; likely to be contaminated with *A. belladonna*, as noted in a batch from a different supplier
Common mallow tea	Canada, 1981–1984 [15]	Two hospitalised cases in 1981 and 1984; packages recovered from patients contained berries of *A. Belladonna*
Marshmallow root tea	Netherlands, 2013 [16,17]	M/27 and his partner (F/28) needed hospital admission; within one week, four other cases (two needed hospital admission) reported to the Dutch National Poisons Information Centre; atropine content 1–10 mg per g; contamination by *A. belladonna*; probably occurred during harvest
Marshmallow root tea	France, 2013 [17]	One case related to the marshmallow root tea purchased in The Netherlands identified via the European Association of Poisons Centres and Clinical Toxicologists network
Lungwort tea	Spain, 2006 [18]	M/76 (two episodes), daughter (F/42, one episode) and granddaughter (F/14, one episode) with anticholinergic toxicity and ED attendance (at least one hospital admission); presence by tropane alkaloids was strongly suspected
Paraguay tea	USA, 1994 [19]	A couple (M/39, F/38), F/20 and a family of four (M/40, F/35, M/18, M/10) presented within three days, requiring ED attendance (*n* = 6) or hospital admission (*n* = 1); contamination with leaves from a plant containing belladonna alkaloids was the most likely explanation, since the samples provided by patients tested positive for atropine, scolopamine, and hyoscyamine
Stinging nettle tea	Austria, 1980 [20]	F/57 needed hospital admission; sample obtained fom patient contained impurities (insects and *A. belladonna* leaves)

^a^ The year of occurrence of the poisoning incident or publication of the report. ED = emergency department.

**Table 2:** Tropane alkaloid poisoning caused by the contamination of *Coleus forskohlii* in slimming pills.

Place, Year [ref]	Details
Italy, 2005 [21]	F/34 was hospitalised after taking galenic product (*C. forskohlii*, green tea, rhodiola, dandelion, hawkweeds)
Italy, 2005 [21]	F/44 was not reported to require hospitalisation or not after taking galenic product (*C. forskohlii*)
Italy, 2005 [21]	F/46, after taking galenic product (*C. forskohlii*)

**Table 3:** Tropane alkaloid poisoning caused by the contamination of one of the prescribed herbs ^a^.

Species	Place, Year ^b^ [ref]	Details
Unknown ^c^	Hong Kong, 1985–1987 [23]	Nine cases (5M, 4F, aged 30–81 years), including one cluster of four cases and one cluster of two cases, admitted to two hospitals; herbal residues available from one case tested positive for atropine; the contaminated herbs were not identified
Unknown ^c^	Hong Kong, 2008–2012 [24]	Five hospitalised cases reported to the Department of Health; herbal samples or herbal residues tested positive for tropane alkaloids; the contaminated herbs were not identified
*Rhizoma Atractylodis*	Hong Kong, 2000–2004 ^d^ [25]	Three hospitalised cases reported to the Department of Health; impurities tested positive for scopolamine and atropine in two; seed-like herb tested positive for scopolamine and atropine in one
*Rhizoma Atractylodis*	Hong Kong, 2002–2011 ^d^ [26]	11 hospitalised cases (3M, 8F, aged 9–52 years, median 45 years) reported to the Department of Health; tropane alkaloids found in four of the suppliers’ samples which came from imports
*Rhizoma Atractylodis*	Ningbo, China, 1999 [27]	Numerous cases reported by several hospitals all related to one batch; found to contain rhizomes of *Scopolia japonica*, which tested positive for atropine and scopolamine
*Rhizoma Atractylodis*	Taizhou, China, 2011 [28]	A cluster of >10 cases (9% of all treated subjects) reported by a health centre; no more new cases after the implicated batch was withdrawn; no impurities found on inspection of samples
*Rhizoma Atractylodis*	Changsha, China, 2011–2012 [29]	Two cases (F/41, F/66) reported by a herbal medicine research centre; found to contain rhizomes of *Solanaceae* plants
*Rhizoma Atractylodis*	Jingmen, China, 2014 [30]	A cluster of cases presented to one hospital; samples obtained from the retailer tested positive for atropine type of alkaloids and contained rhizomes of *H. Niger*
*Radix Aucklandiae*	Hong Kong, 2008–2012 [24]	Two hospitalised cases reported to the Department of Health; sample from the same batch tested positive for tropane alkaloids
*Radix Strobilanthis Forrestii*	Hong Kong, 2010 [31]	One hospitalised case (F/79) reported to the Department of Health; pre-packed sample imported from a herb processing factory tested positive for atropine; atropine, scopolamine, anisodamine detected in patient’s urine
*Rhizoma et Radix Notopterygii*	Hong Kong, 2012 [32]	One hospitalised case (F/63) reported to the Department of Health; herbal residues and sample from herbal shop tested positive for tropane alkaloids

^a^ A *Solanaceae* plant was not prescribed or dispensed, indicating contamination of a non-toxic herb by foreign matters (plant or plant parts) containing tropane alkaloids. So far, there has not been a report of more than one non-toxic herb in any composite herbal formulae having been contaminated; ^b^ The year of occurrence of the poisoning incident or publication of the report; ^c^ The contaminated herb could not be identified; ^d^ The study periods overlapped for 30 months; some of the cases might or might not have been counted twice.
